# α_3_β_4_^∗^ Nicotinic Acetylcholine Receptors Strongly Modulate the Excitability of VIP Neurons in the Mouse Inferior Colliculus

**DOI:** 10.3389/fncir.2021.709387

**Published:** 2021-08-09

**Authors:** Luis M. Rivera-Perez, Julia T. Kwapiszewski, Michael T. Roberts

**Affiliations:** Kresge Hearing Research Institute, Department of Otolaryngology – Head and Neck Surgery, University of Michigan, Ann Arbor, MI, United States

**Keywords:** inferior colliculus, neuromodulation, acetylcholine, nicotinic acetylcholine receptors, auditory system, VIP neurons, pharmacology

## Abstract

The inferior colliculus (IC), the midbrain hub of the central auditory system, receives extensive cholinergic input from the pontomesencephalic tegmentum. Activation of nicotinic acetylcholine receptors (nAChRs) in the IC can alter acoustic processing and enhance auditory task performance. However, how nAChRs affect the excitability of specific classes of IC neurons remains unknown. Recently, we identified vasoactive intestinal peptide (VIP) neurons as a distinct class of glutamatergic principal neurons in the IC. Here, in experiments using male and female mice, we show that cholinergic terminals are routinely located adjacent to the somas and dendrites of VIP neurons. Using whole-cell electrophysiology in brain slices, we found that acetylcholine drives surprisingly strong and long-lasting excitation and inward currents in VIP neurons. This excitation was unaffected by the muscarinic receptor antagonist atropine. Application of nAChR antagonists revealed that acetylcholine excites VIP neurons mainly via activation of α_3_β_4_^∗^ nAChRs, a nAChR subtype that is rare in the brain. Furthermore, we show that acetylcholine excites VIP neurons directly and does not require intermediate activation of presynaptic inputs that might express nAChRs. Lastly, we found that low frequency trains of acetylcholine puffs elicited temporal summation in VIP neurons, suggesting that *in vivo*-like patterns of cholinergic input can reshape activity for prolonged periods. These results reveal the first cellular mechanisms of nAChR regulation in the IC, identify a functional role for α_3_β_4_^∗^ nAChRs in the auditory system, and suggest that cholinergic input can potently influence auditory processing by increasing excitability in VIP neurons and their postsynaptic targets.

## Introduction

Growing evidence indicates that cholinergic signaling through nicotinic acetylcholine receptors (nAChRs) critically shapes sound processing in the central auditory system (Goyer et al., 2016; Askew et al., 2017; Felix et al., 2019; Zhang et al., 2021). The inferior colliculus (IC), the midbrain hub of the central auditory system, receives extensive input from cholinergic neurons in the pontomesencephalic tegmentum (PMT; Motts and Schofield, 2009), and expresses several nAChR subunits, including α_3_, α_4_, α_7_, β_2_, β_3_, and β_4_ (Clarke et al., 1985; Wada et al., 1989; Morley and Happe, 2000; Whiteaker et al., 2002; Salas et al., 2003; Gahring et al., 2004; Happe and Morley, 2004; Bieszczad et al., 2012; Sottile et al., 2017). Because activity in the PMT is influenced by the sleep-wake cycle, attention, rewards, and sensory novelty, it is hypothesized that PMT neurons regulate auditory processing in the IC as a function of behavioral state (Jones, 1991; Kozak et al., 2005; Schofield et al., 2011; Boucetta et al., 2014). Consistent with this, *in vivo* studies have shown that nicotinic drugs alter the gain of input-output functions in IC neurons (Farley et al., 1983; Habbicht and Vater, 1996), and human psychophysics studies indicate that nicotine improves performance in auditory attention and discrimination tasks (Knott et al., 2012; Smucny et al., 2016; Pham et al., 2020), an effect partly attributable to alterations in the IC (Askew et al., 2017). In addition, temporal coding of auditory stimuli is degraded in the IC of α_7_ knockout mice (Felix et al., 2019). However, despite the importance of nAChRs to auditory processing, the cellular mechanisms by which nAChRs influence the excitability IC neurons remain largely unknown.

This gap in knowledge has eluded the field mostly due to the complexity of the neuronal populations in the IC, where it has proven difficult to identify and study specific neuron classes using conventional approaches. We recently overcame this obstacle, identifying vasoactive intestinal peptide (VIP) neurons as the first molecularly identifiable neuron class in the IC (Goyer et al., 2019). Vasoactive intestinal peptide neurons are found throughout the major IC subdivisions, they are glutamatergic, and they have a stellate morphology with spiny dendrites that, within the central nucleus of the IC (ICc), typically extend across two or more isofrequency laminae. Vasoactive intestinal peptide neurons project to several auditory regions, including the auditory thalamus, superior olivary complex, and the contralateral IC, and they receive input from the dorsal cochlear nucleus, the contralateral IC, and likely from other sources. By using the VIP-IRES-Cre mouse model, we can selectively target VIP neurons for electrophysiological and anatomical experiments. Thus, we are in a position for the first time to determine the cellular mechanisms of cholinergic signaling in a defined class of IC neurons.

Here, we hypothesized that the excitability of VIP neurons in the IC is modulated by cholinergic signaling. Using immunofluorescence, we showed that cholinergic terminals are frequently located in close proximity to VIP neurons, suggesting that VIP neurons receive direct cholinergic input. We then found that brief applications of ACh elicited surprisingly long periods of depolarization and spiking in VIP neurons. These responses were not affected by atropine, a muscarinic acetylcholine receptor (mAChR) antagonist, but were largely blocked by mecamylamine, an antagonist partially selective for β_4_-containing receptors, and by SR16584, an antagonist selective for α_3_β_4_^∗^ receptors (^∗^ *indicates that the identity of the fifth subunit in the receptor pentamer is unknown*). Consistent with this, voltage clamp recordings showed that ACh puffs led to prolonged inward currents that were largely blocked by mecamylamine and by SR16584. Moreover, cholinergic responses were resistant to manipulations affecting synaptic transmission, indicating that the nAChRs mediating these responses are expressed by VIP neurons. Finally, we showed that 10 and 30 Hz trains of lower concentration ACh puffs elicited temporal summation in VIP neurons, suggesting that the *in vivo* firing patterns of cholinergic PMT neurons are likely to drive prolonged excitation of VIP neurons. We thus provide the first evidence that α_3_β_4_^∗^ nAChRs, a subtype with limited distribution in the brain, elicit direct and potent excitation of IC VIP neurons. Combined, our data reveal that cholinergic modulation exerts a surprisingly potent and long-lasting increase in the excitability of an important class of IC principal neurons.

## Materials and Methods

### Animals

All experiments were approved by the University of Michigan Institutional Animal Care and Use Committee and were in accordance with NIH guidelines for the care and use of laboratory animals. Animals were kept on a 12-h day/night cycle with *ad libitum* access to food and water. VIP-IRES-Cre mice (*Vip^TM 1(cre)*Zjh*^*/J, Jackson Laboratory, stock # 010908) (Taniguchi et al., 2011) were crossed with Ai14 reporter mice (B6.Cg-*Gt(ROSA)26Sor^TM 14(CAG–tdTomato)Hze^*/J, Jackson Laboratory, stock #007914) (Madisen et al., 2010) to yield F1 offspring that expressed the fluorescent protein tdTomato in VIP neurons. Because mice on the C57BL/6J background undergo age-related hearing loss, experiments were restricted to mice aged P30 – P85, an age range where hearing loss should be minimal or not present (Zheng et al., 1999).

### Immunofluorescence

Mice aged P53 – P85 were deeply anesthetized with isoflurane and perfused transcardially with 0.1 M phosphate-buffered saline (PBS), pH 7.4, for 1 min and then with a 10% buffered formalin solution (Millipore Sigma, cat# HT501128) for 15 min. Brains were collected and post-fixed in the same formalin solution and cryoprotected overnight at 4°C in 0.1 M PBS containing 20% sucrose. Brains were cut into 40 μm sections on a vibratome. Sections were rinsed in 0.1 M PBS, and then treated with 10% normal donkey serum (Jackson ImmunoResearch Laboratories, West Grove, PA) and 0.3% TritonX-100 for 2 h. Slices were incubated overnight at 4°C in rabbit anti-VAChT (3:500, Synaptic Systems, cat# 139103, RRID:AB_887864). This antibody was previously validated by Western blot and has been successfully used to identify cholinergic terminals in the cochlear nucleus and hippocampus (Goyer et al., 2016; Gillet et al., 2018; Zhang et al., 2019). The next day, sections were rinsed in 0.1 M PBS and incubated in Alexa Fluor 647-tagged donkey anti-rabbit IgG (1:500, ThermoFisher, cat# A-31573) for 2 h at room temperature. Sections were then mounted on slides (SouthernBiotech, cat# SLD01-BX) and coverslipped using Fluoromount-G (SouthernBiotech, cat# 0100–01). Images were collected using a 1.40 NA 63x oil-immersion objective and 0.1 μm Z-steps on a Leica TCS SP8 laser scanning confocal microscope.

### Analysis of Cholinergic Terminals Adjacent to VIP Neurons

After immunofluorescence was performed, we used Neurolucida 360 software (MBF Bioscience) to reconstruct VIP neurons and assess the distribution of cholinergic terminals on reconstructed neurons. Terminals that were <2 μm from the dendrites or soma of the reconstructed cell were counted as synapses onto that neuron.

### Brain Slice Preparation

Whole-cell patch-clamp recordings were performed in acutely prepared brain slices from VIP-IRES-Cre x Ai14 mice. Both males (*n* = 40) and females (*n* = 31) aged P30-P50 were used. No differences were observed between animals of different sexes. Mice were deeply anesthetized with isoflurane and then rapidly decapitated. The brain was removed, and a tissue block containing the IC was dissected in 34°C ACSF containing the following (in mM): 125 NaCl, 12.5 glucose, 25 NaHCO_3_, 3 KCl, 1.25 NaH_2_PO_4_, 1.5 CaCl_2_ and 1 MgSO_4_, bubbled to a pH of 7.4 with 5% CO_2_ in 95% O_2_. Coronal sections of the IC (200 μm) were cut in 34°C ACSF with a vibrating microtome (VT1200S, Leica Biosystems) and incubated at 34°C for 30 min in ACSF bubbled with 5% CO_2_ in 95% O_2_. Slices were then incubated at room temperature for at least 30 min before being transferred to the recording chamber. All recordings were targeted at VIP neurons located in the central nucleus of the IC. Because the borders of IC subdivisions are not well-defined in acutely prepared slices, it is possible that a small number of VIP neurons were recorded from just outside the central nucleus, in the dorsal or lateral cortices of the IC.

### Current-Clamp Electrophysiology

Slices were placed in a recording chamber under a fixed stage upright microscope (BX51WI, Olympus Life Sciences) and were constantly perfused with 34°C ACSF at ∼2 ml/min. All recordings were conducted near physiological temperature (34°C). Inferior colliculus neurons were patched under visual control using epifluorescence and Dodt gradient-contrast imaging. Current-clamp recordings were performed with a BVC-700A patch clamp amplifier (Dagan Corporation). Data were low pass filtered at 10 kHz, sampled at 50 kHz with a National Instruments PCIe-6343 data acquisition board, and acquired using custom written algorithms in Igor Pro. Electrodes were pulled from borosilicate glass (outer diameter 1.5 mm, inner diameter 0.86 mm, Sutter Instrument) to a resistance of 3.5 – 5.0 MΩ using a P-1000 microelectrode puller (Sutter Instrument). The electrode internal solution contained (in mM): 115 Kgluconate, 7.73 KCl, 0.5 EGTA, 10 HEPES, 10 Na_2_ phosphocreatine, 4 MgATP, 0.3 NaGTP, supplemented with 0.1% biocytin (w/v), pH adjusted to 7.4 with KOH and osmolality to 290 mmol/kg with sucrose. Data were corrected for an 11 mV liquid junction potential.

To test the effect of ACh on the excitability of VIP neurons, acetylcholine chloride (Sigma cat # A6625), was freshly dissolved each day in a vehicle solution containing (in mM): 125 NaCl, 3 KCl, 12.5 Glucose and 3 HEPES. The solution was balanced to a pH of 7.40 with NaOH. The working concentration of ACh was 1 mM unless stated otherwise. To apply ACh puffs on brain slices, ACh solution was placed in pipettes pulled from borosilicate glass (outer diameter 1.5 mm, inner diameter 0.86 mm, Sutter Instrument) with a resistance of 3.5 – 5.0 MΩ using a P-1000 microelectrode puller (Sutter Instrument) connected to a pressure ejection system built based on the OpenSpritzer design (Forman et al., 2017). The puffer pipette was placed ∼ 20 μm from the soma of the patched cell, and five 10 ms puff applications at 10 psi and 1 min apart were presented per condition. To isolate the receptors mediating the effects of ACh on VIP neurons, we bath applied the following drugs individually or in combination: 1 μM atropine (mAChR antagonist, Sigma), 5 μM mecamylamine (Mec, relatively non-selective antagonist with higher affinity for β_4_ containing receptors, Sigma), 10 μM DHβE (α_4_β_2_^∗^ nAChR antagonist, Tocris), 50 μM SR16584 (α_3_β_4_^∗^ nAChR antagonist, Tocris), and 5 nM methyllycaconitine (MLA, α_7_ nAChR antagonist, Sigma). All drugs were washed-in for 10 min before testing how the drugs affected the responses of the recorded neurons to ACh puffs. In one experiment, antagonists for GABA_*A*_, glycine, AMPA, and NMDA receptors were bath applied to isolate direct effects of ACh on VIP neurons from possible ACh-induced changes in release from terminals synapsing onto VIP neurons. The following drug concentrations were used: 5 μM SR95531 (gabazine, GABA_*A*_ receptor antagonist, Hello Bio), 1 μM strychnine hydrochloride (glycine receptor antagonist, Millipore Sigma), 10 μM NBQX disodium salt (AMPA receptor antagonist, Hello Bio), 50 μM D-AP5 (NMDA receptor antagonist, Hello Bio). All drugs were washed-in for 10 min before testing how the drugs affected the responses of the recorded neurons to ACh puffs. Except for when the effects of atropine alone were directly tested, 1 μM atropine was included in the ACSF under all conditions.

### Effect of Repeated ACh Applications

ACh puffs were applied as described above except at lower concentrations (30 μM and 100 μM). Trials containing trains of 1, 2, 3, 5, or 10 puffs at 10 Hz were delivered with a 1-min intertrial period. Five trials were presented per condition.

### Voltage-Clamp Recordings of nAChR Currents

For voltage-clamp experiments, the recording setup was the same as above except that recordings were performed using an Axopatch 200A amplifier. During the recordings, series resistance compensation was performed using 90% prediction and 90% correction. The series resistance of the electrode was never greater than 10 MΩ. The electrode internal solution contained (in mM): 115 CsOH, 115 D-gluconic acid, 7.76 CsCl, 0.5 EGTA, 10 HEPES, 10 Na_2_ phosphocreatine, 4 MgATP, 0.3 NaGTP, supplemented with 0.1% biocytin (w/v), pH adjusted to 7.4 with CsOH and osmolality to 290 mmol/kg with sucrose. As detailed above, the ACh puffer was placed approximately 20 μm from the soma of the patched cell, and five 10 ms puff applications at 10 psi were presented per condition, waiting 1 min between puffs. Receptor antagonists were applied as described above for the current clamp experiments. 1 μM atropine was included in the ACSF under all conditions. Voltage-clamp holding potentials were not corrected for the liquid junction potential.

### Analysis of Electrophysiological Recordings

Action potential counts and measurements of the area under current clamp depolarizations and voltage clamp currents were made using custom written algorithms in Igor Pro 8 (Wavemetrics). Action potential counts were made with a threshold-crossing algorithm and were verified by eye. To determine the area under current clamp depolarizations, data were first median filtered using a 4000 sample (80 ms) smoothing window to remove action potentials while leaving the waveform of the underlying slow depolarization intact. The area under the median-filtered depolarization was then calculated using the “Area” function. The area under voltage clamp currents was determined by applying the “Area” function to traces that were first median filtered using the same parameters as above. Responses to the five ACh puffs delivered per neuron per treatment condition were averaged, and these average values were used for the summary analyses.

### Statistical Analyses

Data were analyzed and are presented following the estimation statistics approach, which emphasizes effect sizes and their confidence intervals over *p* values (Bernard, 2019; Calin-Jageman and Cumming, 2019). Results from null hypothesis significance tests are also provided, but a focus on the estimation statistics is encouraged. Data analysis and significance tests were performed using custom algorithms combined with the statistical functions available in Igor Pro 8 (Wavemetrics), MATLAB R2021a (MathWorks), and R 4.1.0 (The R Project for Statistical Computing).

In Figures 2–6, comparisons of results are shown using Gardner-Altman estimation plots (two groups) or Cumming estimation plots (three or more groups). The design of these plots was heavily influenced by the DABEST package (Ho et al., 2019), although the plots shown here were made using our own algorithms in MATLAB. Since our data involved repeated measures from individual cells, our estimation plots use parallel coordinates plots to show the measured responses for each cell, with each line representing data from one cell. The parallel coordinates plots are accompanied by paired mean difference plots that show the pairwise differences between control responses and treatment responses. Paired mean differences are presented with bias-corrected and accelerated 95% bootstrap confidence intervals, which were generated using the “boot” package in R using 10,000 resampling iterations (Davison and Hinkley, 1997; Canty and Ripley, 2021). Bootstrap sampling distributions are plotted alongside the mean differences as histograms that were smoothed with the normal kernel function using the MATLAB “fitdist” command.

Statistical tests for differences between two groups were made using the “independence_test” function in the “coin” package in R (Hothorn et al., 2006; Hothorn et al., 2008). The “independence_test” is a non-parametric permutation test based on the theoretical framework of Strasser and Weber (Strasser and Weber, 1999). The “independence_test” was set to use a block design to represent the paired measurements in our data, to perform a two-sided test where the null hypothesis was zero Pearson correlation, and to generate the conditional null distribution using Monte Carlo resampling with 10,000 iterations. Statistical testing for differences between the control group and two or more treatment groups were made using linear mixed models (LMMs) implemented through the “lme4” and “lmerTest” packages in R (Bates et al., 2015; Kuznetsova et al., 2017). Drug treatments were the fixed effects and individual cells were the random effects. *F* statistics, *t* statistics, and *p* values for LMMs were generated using Satterthwaite’s degrees of freedom method as implemented in the “lmerTest” package. In Figure 7, summary data for the responses to trains of acetylcholine puffs are presented as mean ± SD, and the summary data were fit with a linear regression performed in Igor Pro. Test statistics for all significance tests are reported in the figure legends. Effects were considered significant when *p* < 0.05.

## Results

### Cholinergic Synapses Are Found Adjacent to the Somas and Dendrites of VIP Neurons

**FIGURE 1:**
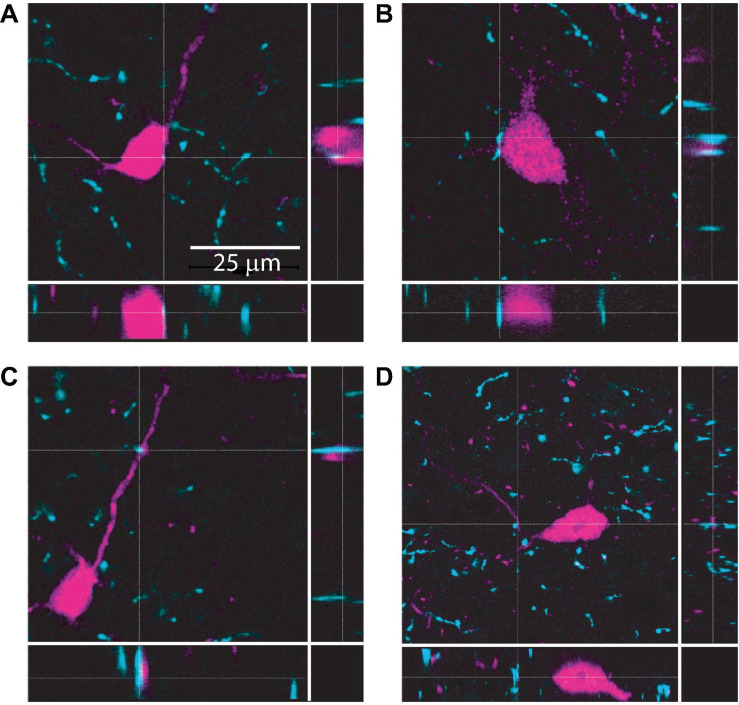
Cholinergic terminals are routinely found in close proximity to VIP neuron somas and dendrites. Confocal images from IC sections show examples of cholinergic terminals (identified by dashed crosshairs) labeled by anti-VAChT (cyan) located <2 μm from the somas (top row, **A,B**) or dendrites (bottom row, **C,D**) of VIP neurons (magenta). The three panels in each image provide a top view and two side views centered on the cholinergic terminal identified by the crosshairs. Images are from 3 mice.

The IC receives cholinergic input from the two nuclei that comprise the PMT: the pedunculopontine tegmental nucleus and the laterodorsal tegmental nucleus. Together, these nuclei distribute cholinergic axons and synapses throughout the IC, contacting both GABAergic and glutamatergic neurons (Motts and Schofield, 2009; Schofield et al., 2011; Noftz et al., 2020; Beebe and Schofield, 2021). However, the specific neuronal populations that cholinergic terminals synapse onto in the IC remain unclear. To test whether VIP neurons receive cholinergic input, we performed immunofluorescence on brain slices from VIP-IRES-Cre x Ai14 mice, in which VIP neurons express the fluorescent protein tdTomato, using an antibody against the vesicular acetylcholine transporter (VAChT). High resolution images were collected using a laser-scanning confocal microscope with a 1.40 NA 63x oil-immersion objective and 0.1 μm Z-steps. Analysis of these images showed that VAChT^+^ boutons and terminals were routinely located <2 μm from the somas, dendrites, or both of VIP neurons (Figure 1). Similar results were observed in IC sections from five mice. These results suggest that VIP neurons receive cholinergic input (Rees et al., 2017). We therefore hypothesized that cholinergic signaling modulates the excitability of VIP neurons.

### Brief Puffs of ACh Drive Prolonged Firing in VIP Neurons *Via* Non-α_7_ nAChRs

**FIGURE 2:**
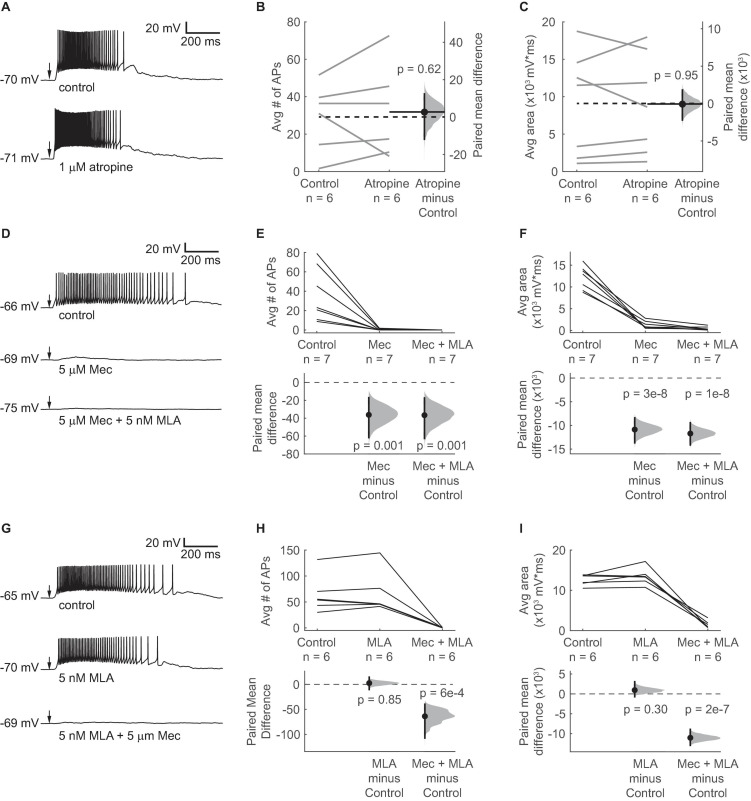
ACh-induced depolarization of VIP neurons is mediated by non-α_7_ nAChRs**. (A)** A 10 ms puff of 1 mM ACh elicited a long-lasting depolarization and spiking in a representative VIP neuron under control conditions (top). 1 μM atropine did not alter the ACh response (bottom). **(B,C)** Atropine did not change the average number of action potentials elicited by ACh or the average area under the median-filtered curve (spikes: paired independence test, *Z* = –0.486, *p* = 0.62, *n* = 6; area: paired independence test, *Z* = 0.086, *p* = 0.95, *n* = 6). Dashed horizontal lines show the mean of the control responses. Solid horizontal lines show the mean difference between the control and atropine conditions. Note that in all remaining experiments, 1 μM atropine was always present in the ACSF. **(D)** Example traces show ACh-elicited depolarization in a VIP neuron in control ACSF (top), 5 μM Mec (middle), and 5 μM Mec + 5 nM MLA (bottom). **(E)** Mec reduced the average number of action potentials elicited by ACh puffs from 36.6 ± 28.2 to 0.4 ± 0.7 (mean ± SD; LMM: treatment effect, *F_2_,_12_* = 11.99, *p* = 0.001, *n* = 7; control vs. Mec, *t*_12_ = –4.22, *p* = 0.001; control vs. Mec + MLA, *t*_12_ = –4.26, *p* = 0.001). **(F)** The total depolarization elicited by ACh puffs, measured as the area under the median filtered curve, was significantly decreased by Mec and further decreased by MLA (LMM: treatment effect *F_2_,_12_* = 111.6, *p* = 2e-8, *n* = 7; control vs. Mec, *t*_12_ = –12.43, *p* = 3e-8; control vs. Mec + MLA, *t*_12_ = –13.39, *p* = 1e-8). **(G)** When MLA was applied before Mec, MLA alone did not have a clear effect on ACh responses. From top to bottom: control, 5 nM MLA, and 5 μM Mec + 5 nM MLA. **(H)** The average number of action potentials elicited by ACh was not significantly affected by MLA, while subsequent application of Mec + MLA eliminated firing (LMM: treatment effect, *F_2_,_10_* = 17.23, *p* = 6e-4, *n* = 6; control vs. MLA, *t*_10_ = 0.20, *p* = 0.85; control vs. Mec + MLA, *t*_10_ = –4.98, *p* = 6e-4). **(I)** MLA alone did not significantly alter the depolarization elicited by ACh, while subsequent application of Mec + MLA caused an 89% reduction in the average depolarization (LMM: treatment effect, *F_2_,_10_* = 120.4, *p* = 1e-7, *n* = 6; control vs. MLA, *t*_10_ = 1.10, *p* = 0.30; control vs. Mec + MLA, *t*_10_ = –12.85, *p* = 2e-7). In **(A,D,G)** arrows indicate the time of the ACh puffs, and voltages indicate resting membrane potential. In **(E,F,H,I)**, horizontal dashed lines indicate the level of zero mean difference, and vertical lines on the paired mean difference points indicate 95% bootstrap confidence intervals.

To test whether acetylcholine alters the excitability of VIP neurons, we targeted current clamp recordings to fluorescent VIP neurons in acute IC slices from VIP-IRES-Cre x Ai14 mice and used a puffer pipette to provide brief puffs of ACh near the recorded cell. We found that 10 ms puffs of 1 mM ACh delivered approximately 20 μm from the VIP cell soma drove depolarization and firing in 116 out of 126 VIP neurons. These effects were surprisingly strong and long-lasting, suggesting that cholinergic signaling can potently increase the excitability of VIP neurons (Figure 2).

Since ACh depolarized VIP neurons for up to 1 s, we first hypothesized that this effect was mediated by a slow metabotropic mechanism involving mAChRs. However, we found that ACh-mediated excitation of VIP neurons was not altered by 1 μM atropine, a mAChR antagonist, indicating that mAChRs are not involved in this phenomenon (Figures 2A–C). For the remainder of this study, all recordings were conducted in the presence of 1 μM atropine, allowing us to isolate effects on nAChRs.

Next, we used mecamylamine (Mec), a broad-spectrum nAChR antagonist partially selective for β4-containing nAChRs (Papke et al., 2008, 2010), and methyllycaconitine (MLA), an antagonist selective for α_7_ nAChRs, to assess the contributions of nAChRs to the cholinergic excitation of VIP neurons. We found that bath application of 5 μM Mec nearly abolished the firing and strongly reduced the depolarization elicited by ACh puffs on VIP neurons. When both 5 μM Mec and 5 nM MLA were applied, the remaining depolarization was nearly eliminated (Figures 2D–F). When 5 nM MLA was applied first, ACh-elicited firing and depolarization in VIP neurons were not significantly altered. Subsequent addition of 5 μM Mec to the bath abolished the ACh effect (Figures 2G–I). Combined, these results suggest that cholinergic modulation of VIP neurons is predominately driven by non-α_7_, Mec-sensitive nAChRs.

### Brief ACh Puffs Elicit a Long-Lasting Inward Current in VIP Neurons

**FIGURE 3:**
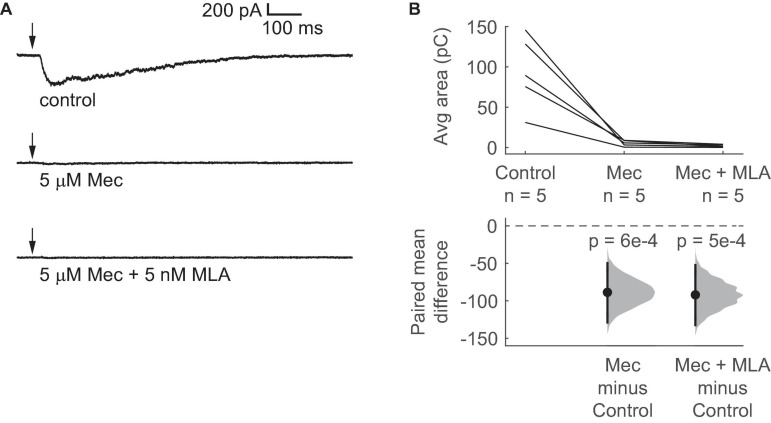
Brief ACh puffs elicit long-lasting inward currents in VIP neurons. **(A)** Example traces of currents elicited by ACh puffs in control ACSF (top), 5 μM Mec (middle), and 5 μM Mec + 5 nM MLA (bottom). Arrows indicate the time of the ACh puffs. **(B)** The total charge flux elicited by ACh, measured by the average area under the median-filtered curve, significantly decreased after bath application of Mec. Subsequent application of MLA did not cause a further reduction (LMM: treatment effect, *F_2_,_8_* = 20.52, *p* = 7e-4, *n* = 5; control vs. Mec, *t*_8_ = –5.44, *p* = 6e-4; control vs. Mec + MLA, *t*_8_ = –5.65, *p* = 5e-4). Horizontal dashed line indicates the level of zero mean difference. Vertical lines on the paired mean difference points indicate 95% bootstrap confidence intervals.

nAChRs are commonly associated with fast, short-lasting depolarizations, but our data suggest that activation of nAChRs elicits prolonged depolarization in VIP neurons. To analyze the currents generated by activation of nAChRs in VIP neurons, we used voltage-clamp recordings with the holding potential at –60 mV. We found that a 10 ms puff of 1 mM ACh elicited an inward current in VIP neurons that lasted hundreds of milliseconds (mean decay τ = 438 ± 173 ms, mean ± SD, based on exponential fit; *n* = 5 neurons; Figure 3A). The peak of the ACh-evoked inward current was –329 ± 154 pA, and the 10 – 90% rise time was 89 ± 31 ms (mean ± SD, *n* = 5 neurons). Furthermore, similar to the depolarizations observed in our current-clamp experiments, 5 μM Mec abolished most of the current elicited by ACh, and the combination of 5 μM Mec and 5 nM MLA abolished the elicited current completely (Figure 3B). Therefore, our results suggest that the nAChRs mediating the effect of ACh on VIP neurons remain activated for extended periods, presumably due to slow kinetics and/or limited desensitization. Since α_7_ nAChRs have fast kinetics and rapid desensitization (Castro and Albuquerque, 1993; Anand et al., 1998; Papke et al., 2000; Mike et al., 2000), both the pharmacology and kinetics of the inward currents observed here are consistent with a mechanism mediated by non-α_7_ nAChRs.

### ACh-Driven Firing in VIP Neurons Does Not Require Activation of Presynaptic nAChRs

**FIGURE 4:**
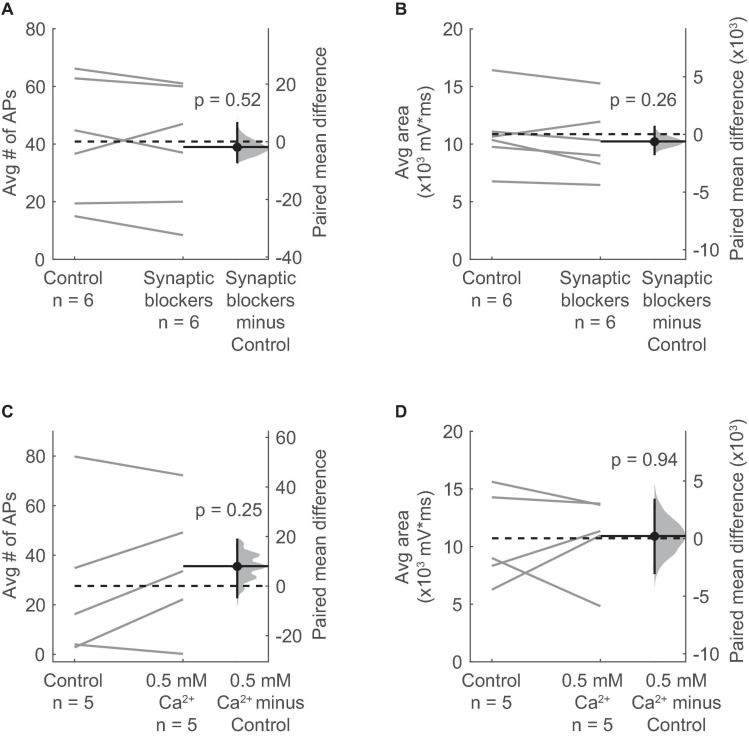
Depolarization of VIP neurons by ACh is consistent with an intrinsic mechanism and not presynaptic effects. **(A,B)** The average number of action potentials **(A)** and depolarization **(B)** elicited by an ACh puff were not affected by bath application of antagonists against AMPA, NMDA, GABA_*A*_, and glycine receptors (10 μM NBQX, 50 μM D-AP5, 5 μM SR95537, and 1 μM strychnine; action potentials: paired independence test, *Z* = 0.724, *p* = 0.52, *n* = 6; area: paired independence test, *Z* = 1.28, *p* = 0.26, *n* = 6). **(C,D)** The average number of action potentials **(C)** and depolarization **(D)** elicited by an ACh puff were unaffected by decreasing the ACSF Ca^2+^ concentration from 1.5 to 0.5 mM (action potentials: paired independence test, *Z* = –1.29, *p* = 0.25, *n* = 5; area: paired independence test, *Z* = –0.137, *p* = 0.94, *n* = 5). **(A–D)** Dashed horizontal lines show the mean of the control responses. Solid horizontal lines show the mean difference between the control and treatment conditions.

Many glutamatergic and GABAergic neurons in the IC express nAChRs (Sottile et al., 2017). In addition, nAChRs are often located on presynaptic terminals where their activation can directly promote neurotransmitter release (Dani and Bertrand, 2007). Therefore, it is possible that the ACh-elicited excitation of VIP neurons requires activation of an intermediate population of neurons or terminals that in turn excite VIP neurons through the release of a different, non-cholinergic neurotransmitter. We therefore tested if cholinergic modulation of VIP neurons requires activation of receptors for glutamate, GABA, and/or glycine, the main neurotransmitters in the IC. By using pharmacology to block these receptors (10 μM NBQX to block AMPA receptors, 50 μM D-APV to block NMDA receptors, 5 μM gabazine to block GABA_*A*_ receptors, and 1 μM strychnine to block glycine receptors), we isolated the effects of ACh puffs on VIP neurons from most other potential inputs. After bath application of the synaptic blockers, we observed that the spiking and depolarization elicited by ACh persisted and was not significantly altered (Figures 4A,B).

Next, we globally reduced synaptic release probability by decreasing the concentration of Ca^2+^ in the ACSF from 1.5 mM to 0.5 mM. Since the relationship between release probability and extracellular Ca^2+^ is described by a power law (Dodge and Rahamimoff, 1967), this reduction in ACSF Ca^2+^ should dramatically decrease neurotransmitter release. We observed that decreasing extracellular Ca^2+^ did not significantly alter the spiking or depolarization elicited by ACh puffs on VIP neurons (Figures 4C,D). These results suggest that ACh acts on nAChRs present on VIP neurons themselves, and not via activation of presynaptic nAChRs.

### α_4_β_2_^∗^ nAChRs Do Not Mediate the Effect of ACh on VIP Neurons

**FIGURE 5:**
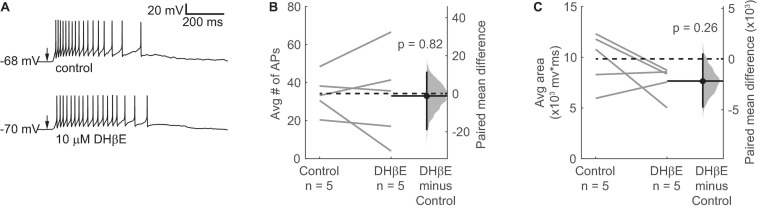
α_4_β_2_* nAChRs do not mediate the ACh-induced depolarization of VIP neurons. **(A)** Example traces show that a spike train elicited by a 10 ms puff of 1 mM ACh (top) was not blocked by 10 μM DHβE (bottom), an α_4_β_2_* nAChR antagonist. Arrows indicate the time of the ACh puffs, and voltages indicate resting membrane potential. **(B,C)** DHβE did not significantly affect the average number of action potentials **(B)** or the total depolarization **(C)** elicited by ACh puffs (action potentials: paired independence test, *Z* = 0.185, *p* = 0.82, *n* = 5; area: paired independence test, *Z* = 1.40, *p* = 0.26, *n* = 5). Dashed horizontal lines show the mean of the control responses. Solid horizontal lines show the mean difference between the control and DHβE conditions.

Our results thus far indicate that Mec-sensitive nAChRs mediate most of the effect of ACh on VIP neurons. However, Mec is a relatively broad-spectrum antagonist of non-homomeric nAChRs, with subtype selectivity depending on the concentration used (Papke et al., 2001, 2008, 2010). Since α_4_β_2_^∗^ nAChRs are widely expressed in the IC and are the most common subtype of nAChR found in the brain (Millar and Gotti, 2009), we performed current-clamp recordings to assess how DHβE, a selective antagonist for α_4_β_2_^∗^ nAChRs, affected the response of VIP neurons to ACh puffs. After bath-applying 10 μM DHβE for 10 min, our results showed that blocking α_4_β_2_^∗^ nAChRs did not significantly alter the spiking or depolarization elicited by ACh application (Figure 5). Therefore, our data suggest that cholinergic modulation of VIP neurons involves little or no contribution from α_4_β_2_^∗^ or α_7_ nAChRs, the most common nAChRs in the brain.

### ACh-Driven Excitation of VIP Neurons Is Mediated by α_3_β_4_^∗^ nAChRs

**FIGURE 6:**
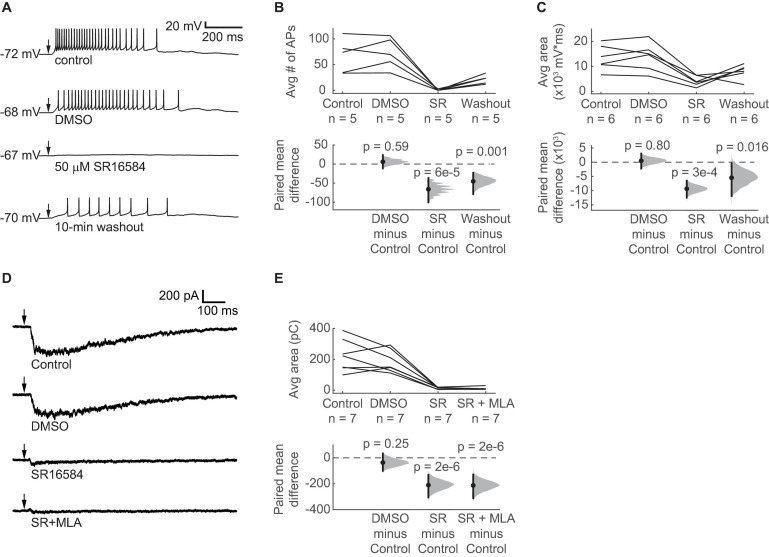
ACh-induced depolarization of VIP neurons is predominately mediated by α_3_β_4_* nAChRs. **(A)** Example traces show that 50 μM SR16584, an α_3_β_4_* nAChR antagonist, blocked action potential firing and most of the depolarization elicited by 1 mM ACh puffs. Conditions from top to bottom: control, vehicle (1:1000 DMSO:ACSF), 50 μM SR16584, and 10-min washout (control ACSF). Arrows indicate the time of the ACh puffs, and voltages indicate resting membrane potential. **(B)** The average number of action potentials elicited by ACh was reduced to 0.64 ± 1.22 (mean ± SD) after bath application of SR16584 (LMM: treatment effect, *F_3_,_12_* = 20.33, *p* = 5e-5, *n* = 5; control vs. DMSO, *t*_12_ = 0.55, *p* = 0.59; control vs. SR, *t*_12_ = –6.01, *p* = 6e-5; control vs. washout, *t*_12_ = –4.13, *p* = 0.001). **(C)** The average total depolarization was also significantly decreased by application of SR16584 (LMM: treatment effect, *F_3_,_15_* = 10.90, *p* = 5e-4, *n* = 6; control vs. DMSO, *t*_15_ = 0.26, *p* = 0.80, control vs. SR, *t*_15_ = –4.64, *p* = 3e-4; control vs. washout, *t*_15_ = –2.71, *p* = 0.016). **(D)** Example traces show that 50 μM SR16584 blocked nearly all of the inward current elicited by 1 mM ACh puffs. Conditions from top to bottom: control, vehicle (1:1000 DMSO:ACSF), 50 μM SR16584, 50 μM SR16584 + 5 nM MLA. Arrows indicate the time of the ACh puffs. Holding potential was –60 mV. **(E)** Inward current elicited by ACh was decreased to 7.4 ± 6.2% (mean ± SD) of control by SR16584 (LMM: treatment effect, *F_3_,_18_* = 27.5, *p* = 6e-7, *n* = 7; control vs. DMSO, *t*_18_ = –1.20, *p* = 0.25; control vs. SR, *t*_18_ = –6.93, *p* = 2e-6; control vs. SR + MLA, *t*_18_ = –7.01, *p* = 2e-6). **(B,C,E)** Horizontal dashed lines indicate the level of zero mean difference. Vertical lines on the paired mean difference points indicate 95% bootstrap confidence intervals.

Although α_3_β_4_^∗^ nAChRs are relatively rare in the brain, previous studies indicate that α_3_ and β_4_ nAChR subunits are expressed in the IC (Wada et al., 1989; Marks et al., 2002, 2006; Whiteaker et al., 2002; Salas et al., 2003; Gahring et al., 2004). In addition, Mec strongly antagonizes α_3_β_4_^∗^ nAChRs at a concentration of 5 μM (Papke et al., 2008, 2010), which we used in our current-clamp and voltage-clamp recordings. We therefore hypothesized that α_3_β_4_^∗^ nAChRs mediate the excitatory effect of ACh on VIP neurons. To test this, we used SR16584, a selective α_3_β_4_^∗^ nAChR antagonist (Zaveri et al., 2010). Because SR16584 is dissolved in DMSO, we first established that a vehicle control (1:1000 DMSO:ACSF) did not affect the ability of 10 ms puffs of 1 mM ACh to excite VIP neurons (Figures 6A–C). Next, we bath applied 50 μM SR16584 and found that it nearly abolished the spiking and strongly reduced the depolarization elicited by ACh (Figures 6A–C), similar to our results with Mec applications. Furthermore, after only a 10-min washout of SR16584, the excitatory effect of ACh partially recovered.

### ACh-Induced Inward Currents in VIP Neurons Are Predominately Mediated by α_3_β_4_^∗^ nAChRs

Based on our current-clamp results, we hypothesized that bath application of SR16584 would abolish most of the inward current elicited by ACh in VIP neurons. To test this, we performed voltage-clamp recordings as described above. As before, ACh elicited large and sustained inward currents that were not altered by the vehicle control (Figure 6D). Application of 50 μM SR16584 abolished 93 ± 6% of the inward current on average (mean ± SD), revealing a much smaller and faster current in 6 of 7 recorded cells, similar to that observed during application of Mec. This remaining current was blocked by application of 5 nM MLA plus 50 μM SR16584, suggesting that it was mediated by α_7_ nAChRs (Figures 6D,E). Together with our current clamp results, these results demonstrate that ACh-induced excitation of VIP neurons is mediated mainly by α_3_β_4_^∗^ nAChRs and provide the first evidence for a functional role of α_3_β_4_^∗^ nAChRs in the IC.

### Repeated ACh Pulses Elicit Temporal Summation in VIP Neurons

**FIGURE 7:**
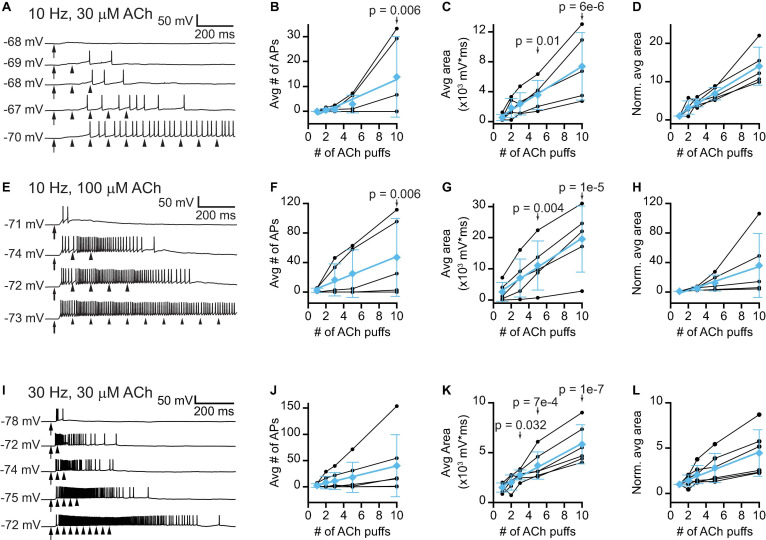
Trains of lower concentration ACh puffs elicit temporal summation and spiking in VIP neurons. **(A)** Example traces show that 10 Hz trains of 10 ms, 30 μM ACh puffs elicited increased depolarization and probability of firing as the train duration increased from 1 to 10 puffs (top to bottom, respectively). **(B)** In 3 out of 5 neurons, increasing the number of 30 μM ACh puffs led to spiking. LMM analysis of the responses of all 5 neurons revealed a significant difference in response between 10 puffs and 1 puff (LMM: treatment effect, *F_4_,_16_* = 3.61, *p* = 0.028, *n* = 5; 1 puff vs. 2 puffs, *t*_16_ = 0.11, *p* = 0.91; 1 puff vs. 3 puffs, *t*_16_ = 0.19, *p* = 0.86; 1 puff vs. 5 puffs, *t*_16_ = 0.68, *p* = 0.51; 1 puff vs. 10 puffs, *t*_16_ = 3.19, *p* = 0.006). **(C,D)** In 5 out of 5 neurons, increasing the number of 30 μM ACh puffs progressively increased the absolute **(C)** or normalized **(D)** total depolarization, measured as the area under the median-filtered curve, indicating that temporal summation occurred (LMM of absolute values: treatment effect, *F_4_,_16_* = 12.8, *p* = 7e-5, *n* = 5; 1 puff vs. 2 puffs, *t*_16_ = 1.23, *p* = 0.24, 1 puff vs. 3 puffs, *t*_16_ = 1.77, *p* = 0.10; 1 puff vs. 5 puffs, *t*_16_ = 2.92, *p* = 0.01; 1 puff vs. 10 puffs, *t*_16_ = 6.63, *p* = 6e-6). Cyan data in **(B–D)** show mean ± SD responses and linear fits to these means [**(B)**, slope = 1.6 APs/puff, *r* = 0.97; **(C)**, slope = 730 mV*ms/puff, *r* = 0.99; **(D)**, slope = 1.4 normalized units/puff, *r* = 0.99]. **(E)** Example traces show that 10 Hz trains of 10 ms, 100 μM ACh puffs elicited increased depolarization and probability of firing as the train duration increased from 1 to 10 puffs (top to bottom, respectively). **(F)** In 4 out of 5 neurons, increasing the number of 100 μM ACh puffs led to spiking. LMM analysis of the responses of all 5 neurons revealed a significant difference between 10 puffs and 1 puff (LMM: treatment effect, *F_3_,_12_* = 3.99, *p* = 0.03, *n* = 5; 1 puff vs. 3 puffs, *t*_12_ = 1.08, *p* = 0.30; 1 puff vs. 5 puffs, *t*_12_ = 1.72, *p* = 0.11; 1 puff vs. 10 puffs, *t*_12_ = 3.38, *p* = 0.006). **(G,H)** In 5 out of 5 neurons, increasing the number of 100 μM ACh puffs progressively increased the absolute **(G)** or normalized **(H)** total depolarization, measured as the area under the median-filtered curve, indicating that temporal summation occurred (LMM of absolute values: treatment effect, *F_3_,_12_* = 18.0, *p* = 9.7e-5, *n* = 5; 1 puff vs. 3 puffs, *t*_12_ = 1.87, *p* = 0.09; 1 puff vs. 5 puffs, *t*_12_ = 3.54, *p* = 0.004; 1 puff vs. 10 puffs, *t*_12_ = 7.07, *p* = 1e-5). Cyan data in **(F–H)** show mean ± SD responses and linear fits to these means [**(F)**, slope = 4.8 APs/puff, *r* = 0.99; **(G)**, slope = 1860 mV*ms/puff, *r* = 0.99; **(H)**, slope = 4.0 normalized units/puff, *r* = 0.99]. **(I)** Example traces show that 30 Hz trains of 10 ms, 30 μM ACh puffs elicited increased depolarization and probability of firing as the train duration increased from 1 to 10 puffs (top to bottom, respectively). **(J)** In 4 out of 6 neurons, increasing the number of 30 μM ACh puffs led to increased spiking. However, LMM analysis did not reveal a significant effect of puff number (LMM: treatment effect, *F_4_,_20_* = 2.70, *p* = 0.06, *n* = 6). **(K,L)** In 6 out of 6 neurons, increasing the number of 30 μM ACh puffs progressively increased the absolute **(K)** or normalized **(L)** total depolarization, measured as the area under the median-filtered curve, indicating that temporal summation occurred (LMM of absolute values: treatment effect, *F_4_,_20_* = 19.5, *p* = 1e-6, *n* = 6; 1 puff vs. 2 puffs, *t*_20_ = 0.92, *p* = 0.37; 1 puff vs. 3 puffs, *t*_20_ = 2.31, *p* = 0.032; 1 puff vs. 5 puffs, *t*_20_ = 4.02, *p* = 7e-4; 1 puff vs. 10 puffs, *t*_20_ = 7.91, *p* = 1e-7). Cyan data in **(J–L)** show mean ± SD responses and linear fits to these means [**(J)**, slope = 4.1 APs/puff, *r* = 0.99; **(K)**, slope = 480 mV*ms/puff, *r* = 0.99; **(L)**, slope = 0.38 normalized units/puff, *r* = 0.99]. In **(A,E,I)** arrows and arrowheads indicate the times of ACh puffs, and voltages indicate resting membrane potential. LMM analysis was not run for the normalized data in **(D,H,L)** since LMM results for the non-normalized data are provided in **(C,G,K)**.

Thus far we have examined how isolated puffs of 1 mM ACh affected the excitability of VIP neurons. However, the time course and concentration of ACh released from cholinergic synapses onto VIP neurons *in vivo* is unknown. Previous studies show that the average firing rates of cholinergic PMT neurons *in vivo* tend to be rather low, typically less than a few Hz (Boucetta et al., 2014), but arousing sensory stimuli elicit brief bursts of firing that can reach 100 – 200 Hz (Reese et al., 1995a,b; Sakai, 2012). In addition, our immunofluorescence data suggest that VIP neurons often receive multiple cholinergic inputs, which may reflect convergence from multiple PMT neurons. We therefore decided to test the effects of 10 and 30 Hz trains of ACh puffs, reasoning that VIP neurons would likely encounter these frequencies of input *in vivo*. Based on the slow kinetics and limited desensitization of α_3_β_4_^∗^ nAChRs (David et al., 2010), we hypothesized that lower concentrations of ACh delivered in trains would elicit long-lasting excitation of VIP neurons due to temporal summation of cholinergic EPSPs. To test this, we made current-clamp recordings from VIP neurons while delivering trains of 1 – 10 puffs of 30 μM or 100 μM ACh at 10 Hz (Figures 7A,E) or 30 μM ACh at 30 Hz (Figure 7I). We observed that as the number of puffs increased, VIP neurons increasingly depolarized and could transition from firing no spikes in response to a single ACh puff to firing trains of spikes in response multiple ACh puffs (Figures 7B,F,J). The amount of depolarization elicited by increasing numbers of ACh puffs, as measured by the average area under the median-filtered trace, produced rising input-output functions (Figures 7C,G,K). Linear fits to the means of the normalized responses for 10 Hz trains had slopes of 1.4 and 4.0 normalized units/puff, indicating that temporal summation was supralinear on average for both 30 μM and 100 μM puff trains, respectively (cyan data, Figures 7D,H). The 30 Hz trains of 30 μM ACh puffs resulted in sublinear integration, with a linear fit to the means of the normalized responses having a slope of 0.38 normalized units/puff (cyan data, Figure 7L). Although this relationship was sublinear, the slope was positive, and temporal summation still occurred, with the total depolarization after 10 puffs being 3.8x that elicited by a single puff.

Together, these results suggest that even if cholinergic synapses *in vivo* elicit smaller and/or briefer EPSPs, these EPSPs will be subject to temporal summation during periods of heightened PMT activity, thereby driving excitation of VIP neurons more effectively than isolated inputs. In addition, our results point to a potentially complicated relationship between the frequency of cholinergic input trains and the extent of temporal summation, suggesting that different patterns of cholinergic input may support diverse computations in VIP neurons. A more comprehensive analysis of the relationship between cholinergic input frequency and temporal summation in VIP neurons awaits a future study when cholinergic inputs to VIP neurons can be directly stimulated, most likely with optogenetics, over a wider range of frequencies. The present results suggest that specific patterns of cholinergic excitation can combine in diverse ways with the ascending and local auditory inputs that VIP neurons receive (Goyer et al., 2019) to critically reshape auditory processing in VIP neurons and their postsynaptic targets.

## Discussion

Here, we report the first cellular-level mechanism for cholinergic modulation in the auditory midbrain. Our data show that cholinergic terminals are routinely found in close proximity to the dendrites and somas of VIP neurons in the IC. In whole-cell recordings, brief applications of ACh to VIP neurons elicited surprisingly strong, long-lasting depolarizations and sustained inward currents. Despite the prolonged nature of these responses, they were not altered by blocking muscarinic receptors. Instead, using several nAChR antagonists, we determined that ACh excites VIP neurons mainly by activating α_3_β_4_^∗^ nAChRs, with a small contribution from α_7_ nAChRs. α_3_β_4_^∗^ nAChRs are rare in the brain and have mainly been studied in the medial habenula and interpeduncular nucleus, where they play an important role in nicotine addiction (Sheffield et al., 2000; Grady et al., 2009; Scholze et al., 2012; Beiranvand et al., 2014). Our results uncover a novel role for α_3_β_4_^∗^ receptors in the central auditory pathway, revealing a potent neuromodulatory mechanism in which ACh can drive a sustained increase in the excitability of VIP neurons. Since VIP neurons project locally, to the auditory thalamus, and to several other auditory and non-auditory brain regions, cholinergic modulation of VIP neurons has the potential to exert widespread influence on auditory processing and its downstream effects.

### α_3_β_4_^∗^ nAChRs Mediate Prolonged Depolarization of VIP Neurons

Although nAChRs are often noted for driving fast and brief responses to cholinergic inputs, these effects are generally attributable to α_7_ nAChRs, which have fast kinetics and rapid desensitization (Christophe et al., 2002; Arroyo et al., 2012; Bennett et al., 2012). A growing number of studies have documented instances in which non-α_7_ nAChRs mediate longer-lasting changes in neuronal excitability. For example, in VIP interneurons in the auditory cortex, nicotine induces firing for up to several minutes, and this effect was blocked by DHβE, an α_4_β_2_^∗^ nAChR antagonist (Askew et al., 2019). In layer 1 of cerebral cortex, α_7_ nAChRs mediate an early, fast response to ACh, while non-α_7_ nAChRs mediate a later, slower response (Christophe et al., 2002; Arroyo et al., 2012; Bennett et al., 2012). Likewise, at the motoneuron-Renshaw cell synapse in the spinal cord, in combination with glutamatergic signaling, homomeric α_7_ nAChRs mediate an early, fast response to ACh, while α_4_β_2_^∗^ nAChRs mediate a slower, longer-lasting response (Lamotte d’Incamps and Ascher, 2008; d’Incamps et al., 2012; Lamotte d’Incamps et al., 2018).

While previous studies show that mRNA for α_7_, α_4_, and β_2_ nAChR subunits is common in the IC (Clarke et al., 1985; Wada et al., 1989; Morley and Happe, 2000; Happe and Morley, 2004; Bieszczad et al., 2012; Sottile et al., 2017), our data point to a limited role for α_7_ and no functional role for α_4_β_2_^∗^ nAChRs in VIP neurons. Instead, we found that α_3_β_4_^∗^ nAChRs are the dominant nAChRs in VIP neurons, mediating a strong, long-lasting depolarization in response to ACh application. Consistent with our observations, α_3_β_4_^∗^ nAChRs are capable of mediating sustained currents due to their slow desensitization, with time constants on the order of seconds, and relatively long single-channel open times and burst durations (David et al., 2010). In addition, *in situ* hybridization studies and binding studies have consistently shown that the IC is one of the few places in the brain where α_3_ and β_4_ nAChRs are expressed (Wada et al., 1989; Marks et al., 2002, 2006; Whiteaker et al., 2002; Salas et al., 2003; Gahring et al., 2004), and β_4_ knockout mice exhibit decreased α_3_ mRNA levels in the IC, supporting the hypothesis that α_3_ and β_4_ subunits interact in the IC (Salas et al., 2004). Our findings confirm and extend these results by providing the first evidence of a functional role for α_3_β_4_^∗^ nAChRs in the IC. Moreover, the widespread expression of α_3_ and β_4_ mRNA observed in past *in situ* hybridization studies suggests that other IC neuron types, in addition to VIP neurons, are likely to express α_3_β_4_^∗^ nAChRs. Application of the pharmacological approach used here to additional neuron types will help expand our understanding of the functional roles of α_3_β_4_^∗^ nAChRs in the IC.

It is important to note that α_3_β_4_^∗^ nAChRs can have two stoichiometries, (α_3_β_4_)_2_α_3_ or (α_3_β_4_)_2_β_4_, and can also combine with a different fifth subunit, (α_3_β_4_)_2_X, where the fifth subunit can be α_2_, α_5_, α_6_, or β_3_ (Scholze and Huck, 2020). Previous studies indicate that the exact subunit composition of an α_3_β_4_^∗^ nAChR has some effect on Ca^2+^ permeability and desensitization rate, but generally little or no effect on the potency and efficacy of ACh (Wang et al., 1996; Gerzanich et al., 1998; Groot-Kormelink et al., 2001; Papke et al., 2010; Stokes and Papke, 2012). It will be important for future studies to determine which type or types of α_3_β_4_^∗^ nAChRs are expressed in VIP neurons.

### Trains of Cholinergic Inputs May Drive Long-Lasting Modulatory Effects

To mimic more *in vivo*-like patterns of cholinergic input, we tested how VIP neurons responded to trains of ACh puffs. Our results show that, even at a relatively low application rate of 10 Hz, cholinergic EPSPs underwent substantial temporal summation in VIP neurons. This temporal summation allowed 10 and 30 Hz trains of 30 μM ACh puffs to transition from eliciting no spikes with 1 or 3 puffs to multiple spikes with 5 or 10 puffs. Trains of 100 μM ACh puffs elicited an even more pronounced increase in firing. Although ACh puffs probably do not match the concentration and time course of synaptically released ACh *in vivo*, our results show that ACh strongly excited VIP neurons under a range of conditions, uncovering a cellular mechanism that likely drives similar effects *in vivo* and highlighting the need for future experiments to build on these results.

It is well established that cholinergic PMT neurons, the source of cholinergic input to the IC, alter their firing rate as a function of behavioral state. For example, Boucetta et al. found that cholinergic PMT neurons fired maximally during the active wake and paradoxical sleep states (mean firing rates of 2.3 Hz and 3.7 Hz, respectively) and nearly ceased firing during slow wave sleep (0.04 Hz) (Boucetta et al., 2014). Sakai found similar changes across the sleep-wake cycle, but also found that arousing stimuli (a hand clap or an air puff) drove cholinergic PMT neurons to fire bursts of 2 – 5 spikes with instantaneous frequencies of 100 – 200 Hz (Sakai, 2012). Many PMT neurons also respond to sensory stimuli. For example, almost half of PMT neurons fire in response to auditory click stimuli, with half of these neurons firing short latency bursts (Reese et al., 1995a,b). Such responses might be driven by the primary auditory cortex, which projects to the PMT (Schofield and Motts, 2009; Schofield, 2010). Furthermore, our immunofluorescence data suggest that VIP neurons often integrate multiple cholinergic inputs. It therefore seems likely that certain behavioral states and sensory stimuli drive cholinergic input to VIP neurons at rates sufficient to elicit the temporal summation we observed. Thus, our data support the hypothesis that cholinergic input from the PMT drives prolonged increases in the excitability in VIP neurons as a function of behavioral state and sensory input. Since VIP neurons project broadly within and beyond the IC, changes that alter cholinergic input to VIP neurons have the potential to drive wide-ranging changes in the excitability of the auditory and non-auditory circuits that VIP neurons target.

### Functional Implications for Auditory Processing

Previous studies have identified clear roles for muscarinic signaling in the IC, including roles in cortically driven plasticity (Ji et al., 2001; Ji and Suga, 2009) and stimulus specific adaptation (Ayala and Malmierca, 2015), but the roles of nicotinic signaling in the IC are less clear. Psychophysical studies indicate that systemic nicotine exposure in non-smokers can enhance performance in auditory tasks (Harkrider and Hedrick, 2005; Knott et al., 2009; Pham et al., 2020). Intriguingly, recent work from Askew and colleagues suggests that systemic nicotine sharpens frequency tuning in the IC, which likely contributes to sharper tuning in auditory cortex and improved discrimination in behavioral tasks (Askew et al., 2017). However, the authors found that nicotine mainly suppressed activity in the IC. Since we found that activation of nAChRs increases VIP neuron excitability, this raises the interesting possibility that the local projections of VIP neurons might mainly target inhibitory neurons. Alternatively, a more complicated circuit interaction might occur. We plan to test how cholinergic modulation of VIP neurons shapes the auditory response properties of VIP neurons and their postsynaptic targets in future studies.

Finally, it is unknown how cholinergic signaling shapes the excitability of other IC neuron classes. The IC contains a rich diversity of neurons, but these have long proved difficult to reliably classify, making it difficult to investigate cholinergic modulation in a systematic way. Fortunately, in addition to VIP neurons, recent studies have identified GABAergic NPY neurons (Silveira et al., 2020) and glutamatergic CCK neurons (Kreeger et al., 2021) as distinct IC neuron classes. To gain a fuller understanding of how cholinergic modulation shapes auditory processing in the IC, it will be important to determine the diverse effects cholinergic modulation exerts on these and other, yet to be identified, neuron classes.

## Data Availability Statement

The raw data supporting the conclusions of this article will be made available by the authors, without undue reservation.

## Ethics Statement

The animal study was reviewed and approved by the University of Michigan Institutional Animal Care and Use Committee.

## Author Contributions

LR-P and MR conceived of the study, obtained funding, and prepared the initial draft of the manuscript. LR-P and JK performed experiments. MR supervised the study. All authors designed experiments, analyzed data, prepared figures, revised the manuscript, and approved the submitted version.

## Conflict of Interest

The authors declare that the research was conducted in the absence of any commercial or financial relationships that could be construed as a potential conflict of interest.

## Publisher’s Note

All claims expressed in this article are solely those of the authors and do not necessarily represent those of their affiliated organizations, or those of the publisher, the editors and the reviewers. Any product that may be evaluated in this article, or claim that may be made by its manufacturer, is not guaranteed or endorsed by the publisher.
